# A measure of inconsistencies in intertemporal choice

**DOI:** 10.1371/journal.pone.0224242

**Published:** 2019-10-30

**Authors:** Salvador Cruz Rambaud, Isabel González Fernández

**Affiliations:** Departamento de Economía y Empresa, Universidad de Almería, Almería, Spain; Shandong University of Science and Technology, CHINA

## Abstract

The aim of this paper is to derive an index able to indicate if a discount function exhibits increasing or decreasing impatience, and, even, in the last case, whether the decreasing impatience is moderate or strong. Moreover, it will be shown that the sign of this indicator coincides with the sign of the convexity index of the discount function when only considering the cases of increasing and decreasing impatience. Consequently, this parameter supposes an improvement of Prelec’s index of convexity. The main advantage of this novel measure is that, the same as Prelec’s index, it uses the differential calculus and, moreover, can be easily plotted by showing the changes from a type of impatience to another one according to time.

## Introduction

Intertemporal choice refers to the process of decision-making between several options whose monetary amounts occur at different moments of time. It can be treated by means of a discount function [1] which measures the present value of a dated reward, or alternatively by means of a preference relation ⪰ (at least as preferred as) over a set of outcomes *X* × *T*. The most famous representation theorem of preferences was provided by Fishburn and Rubinstein [2]: If order, monotonicity, continuity, impatience, and separability hold, and *X* is an interval, then there are continuous real-valued functions *u* on *X* and *F* on *T* such that

$$\begin{array}{c} (x,s)⪰(y,t)\;\text{if,}\;\text{and}\;\text{only}\;\text{if,}\;u(x)F(s)\geq u(y)F(t), \end{array}$$ (1)

where *u*(*x*) (called the *utility*) is increasing, *u*(0) = 0, and *F*(*t*) (called the *unitary discount function*) is decreasing and positive.

In the field of economics, the modelization of intertemporal choice started when Samuelson [3] proposed the Discounted Utility (DU) Model. The foundation of this model is the use of a constant instantaneous discount rate which leads to a consistent choice behavior represented by exponential discounting. In other words, Samuelson’s model supposes that people act in such a way that their decisions do not vary with the passage of time. However, some recent studies in the field of behavioral finance and neuroeconomics have revealed the presence of certain limitations, called *anomalies*, shown by the DU Model.

Nevertheless, before starting with the contents of this paper, we have to recall that the analysis of intertemporal choice process revolves around the concept of impatience. Several authors (e.g., [4]) have defined this key concept as a synonym of *impulsivity*, i.e., a strong preference for small immediate rewards over large delayed ones. From another point of view, if *F* is a discount function, the *impatience* associated to the interval [*t*_1_, *t*_2_] was defined by [1] as 1 minus the value of the discount ratio, *f*(*t*_1_, *t*_2_), corresponding to this interval. Specifically:

$$\begin{array}{c} 1-f\left(t_{1},t_{2}\right)≔1-\frac{F(t_{2})}{F(t_{1})}=1-\exp\{-\int_{t_{1}}^{t_{2}}\delta \left(x\right)dx\}, \end{array}$$ (2)

where *δ*(*x*) is the instantaneous discount rate at time *x*, defined as:

$$\begin{array}{c} \delta \left(x\right)≔-\frac{d\ln F(z)}{dz}{|}_{z=x}=-\frac{F^{\prime }\left(x\right)}{F(x)}. \end{array}$$ (3)

From this equation the following observations can be pointed out:

- The so-defined impatience lies in the interval [0, 1].
- The degree of *patience* of an individual can be measured by the discount factor. In effect, the lesser the discount factor in [*t*_1_, *t*_2_], the more sloped is the discount function in such interval, and then the greater is the preference for immediate over delayed rewards, i.e., people are less patient (more impatient).
- The instantaneous discount rate represents the impatience of a decision-maker at a given moment.

This manuscript has been focused on the analysis of inconsistency in intertemporal choice, that is to say, on the existence of preference reversals, which means that the subject changes his/her initial choice decision when the offered rewards are delayed over the same period of time. Several economists (such as [5, 6]), based on [7], defined the reversal of preferences as a discrepancy between the current decision-making and the same choice in the future. To explain this phenomenon, it is very useful the following example where [5] required people to choose between a small but earlier reward and a larger but later reward. After taking his/her decision on the small earlier reward, these scholars delayed both amounts preserving the temporal interval between them. Now, some decision-makers turned their preference towards the larger later reward, even for very small amounts of added delay. Observe that the concept of *time inconsistency* agglutinates the idea of impulsivity and self-control. This manuscript will be devoted to the analysis of the delay effect or time inconsistency by providing a novel measurement of the degree of inconsistency in the ambit of intertemporal choice.

Taking into account that the inconsistency shown by a subject can be described by the variation of the instantaneous discount rate, this anomaly could be explained as the variation of time preference [8]. In particular, decreasing (resp. increasing) impatience is equivalent to require a decreasing (resp. increasing) instantaneous discount rate [9].

In order to design a new measure of inconsistency, consider the indifference relation (*γ*, *s*) ∼ (*β*, *t*), with *s* < *t*, called an *indifference pair*. If the availability of the reward *β* is delayed until moment *t* + *τ*, with *τ* > 0, the delay *σ* > 0 for which the former indifference relation is preserved, (*γ*, *s* + *σ*) ∼ (*β*, *t* + *τ*), satisfies [10]:

$$\begin{array}{c} \lim_{\tau \to 0}\frac{\sigma }{\tau }=\frac{\delta (t)}{\delta (s)}. \end{array}$$ (4)

This equation represents the relative instantaneous variation in the availability of rewards which will be denoted by *v*(*s*, *t*) and will be called the *instantaneous variation rate*. Obviously, it can be derived that time preference exhibits decreasing impatience if, and only if, the instantaneous variation rate is less than one.

On the other hand, [11] considered that the degree of inconsistency can be represented by the convexity index of the logarithm of the discount function. The drawback of this indicator is the difficulty of its measure, so [12, 13] introduced two novel measures of decreasing impatience, viz the hyperbolic factor and the DI-index. The main advantage is that both measures can be calculated starting from experimental data without any knowledge of utility, so they can also be used when preferences cannot be represented by a discounted utility, unlike Prelec’s measure which requires the representation of preferences by means of a discount function. First, Rohde [12], starting from two indifference pairs, (*γ*, *s*) ∼ (*β*, *t*) and (*γ*, *s* + *σ*) ∼ (*β*, *t* + *τ*), where *γ* and *β* ≻ 0, with *s* < *t*, *σ* > 0 and *τ* > 0, proposed the so-called *hyperbolic factor* as the function defined by:

$$\begin{array}{c} H\left(s,t,\sigma ,\tau \right)≔\frac{\tau -\sigma }{t\sigma -s\tau }. \end{array}$$

Starting from the hyperbolic factor, Rohde [12] defined increasing impatience, moderately decreasing impatience and strongly decreasing impatience (see Section 2). However, the hyperbolic factor is a measure of impatience only for people who exhibit moderately decreasing impatience or increasing impatience.

Consequently, Rohde [13] provided another measure of decreasing impatience, the so-called *DI-index*, defined by:

$$\begin{array}{c} \text{DI-index}=\frac{\tau -\sigma }{\sigma (t-s)}, \end{array}$$ (5)

which is an approximation of Prelec’s degree of inconsistency, *P*(*t*). In effect, it can be shown [14] that

$$\begin{array}{c} \underset{\begin{array}{c} \tau \to 0\\ s\to t \end{array}}{\text{lim}}\text{DI-index}=P\left(t\right). \end{array}$$ (6)

The DI-index does not have the aforementioned problem and can also be computed for people who exhibit strongly decreasing impatience. Moreover, as indicated, the DI-index approximates Prelec’s measure of inconsistency, whilst the hyperbolic factor does not. Table 1 summarizes the sign of the measures related with inconsistencies in intertemporal choice.

**Table 1:** Different measures of inconsistency. Source: Own elaboration.

Types of inconsistency	*δ*′(*t*)	*H*(*s*, *t*, *σ*, *τ*)	DI-index and *P*(*t*)
Strongly decreasing impatience	−	−	+
Moderately decreasing impatience	−	+	+
Increasing impatience	+	−	−

Observe that, in order to know if an intertemporal choice exhibits (strongly or moderately) decreasing or increasing impatience, we have to calculate the sign of at least two indexes in Table 1. Consequently, the objective of this manuscript is to improve these measures by obtaining a new index able to detect all the aforementioned types of impatience.

Finally, let us provide more justification about the need of introducing the concepts of strongly and moderately decreasing impatience. From a managerial point of view, [15] point out that managers in private companies or public institutions may be affected by stress because their decisions about long-term business strategies can be in conflict with short-term profits required by the company. This is an important issue since stress may give rise to inconsistency in their decisions involving intertemporal choices. From a pharmacological perspective, in a recent article, [16] stated that drug addicts and people with other diseases such as obesity, gambling addiction, attention deficit hyperactivity disorder and schizophrenia, discount future stimuli more quickly than those people who do not have these addictions or diseases. It could be said that these people are more impulsive or impatient than individuals without addiction or these diseases. After a wide revision of the literature on addictive behavior and the discount of future rewards [17], a strong evidence of greater discount and even preference reversals was found in people with addictive behavior. There are empirical studies where only monetary rewards (mainly hypothetical) are discounted, but there are also empirical works which compare the discount of monetary and non-monetary rewards by people suffering or not addictive behavior. The same as non-monetary rewards, hypothetical amounts of cigarettes [18, 19], heroin [20], crack/cocaine [21] and alcohol [22] have been used. As a general conclusion, all these addictive substances were discounted more than money by their users. In addition, the largest discount was applied to the monetary rewards of drug users.

The consideration of this so-called “excessive discount” [16] and the reversion of preferences, as processes which underlie diseases and other disorders, justify the necessity of distinguish the impatience showed by a discount function between moderate and strong. In [23], the discount function $F\left(t\right)=\frac{1}{1+it^{k}}$ (*i* > 0 and *k* > 0) was proposed as an alternative to hyperbolic discounting in order to describe the excessive discount revealed by [16]. Unfortunately, this discount function does not show strongly decreasing impatience whereby, in Section 4, we will present a methodology to obtain discount functions exhibiting all types of impatience by distorting a discount function which exhibits decreasing impatience with the deformation *D*(*t*) = *t*^*k*^ (*k* > 0).

**Fig 1:**
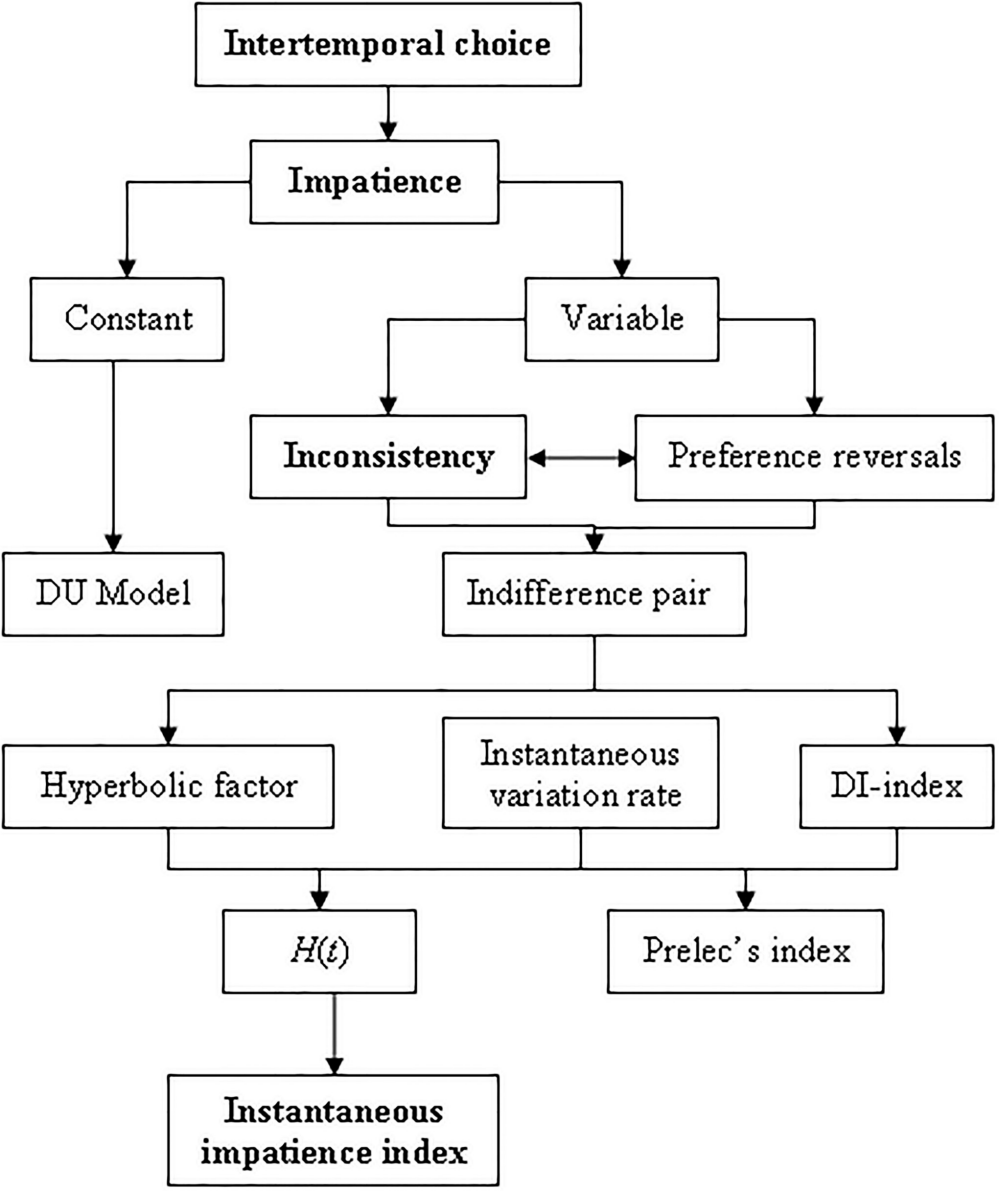
Structure of sections 1 and 3. Source: Own elaboration.

Fig 1 summarizes the content of Section 1.

This paper has been organized as follows. In the Introduction, we have centered the objective of this manuscript by indicating its justification and the main previous measures of inconsistency. Thus, we are mentioned the measure proposed by Prelec [11], and the hyperbolic factor and the DI-index both proposed by Rohde [12, 13]. In Section 2, we have characterized all types of inconsistencies starting from two indifference pairs by using two approaches: one based on preferences and the other one quantified by a discount function. In Section 3, we have proposed a new measure of inconsistency, denoted by *I*^3^(*t*), starting from the concepts of elasticity and hyperbolic factor. This novel tool will allow us to distinguish between all types of impatience according to its sign and its possible values. In this section, we will characterize the *q*-exponential discounting by demonstrating that, in this and only in this case, *I*^3^(*t*) is constant. In Section 4, we have analyzed the behavior of this new measure of inconsistency in the case of S-inverse discount functions, by considering the particular case of deforming a CRDI discount function. Finally, Section 5 summarizes and concludes.

## Variation of impatience

As indicated in the Introduction, the objective of this manuscript is to improve the measures included in Table 1 by obtaining a new index able to detect all types of impatience. This novel index will be derived from the limit of the hyperbolic factor when *τ* → 0 and *s* → *t*, and will be computed as 1 plus the ratio of the derivative of the instantaneous discount rate to the derivative of the elasticity of the corresponding differentiable discount function, this quotient being powered to the sign of *δ*′(*t*) + *ϵ*′(*t*) (see Section 3). Particularly, in this paper we will focus on the S-inverse discount functions [24, 25] which exhibit first increasing and then decreasing impatience. This type of discount function can be obtained by deforming [26] a discount function which exhibits decreasing impatience with the Stevens’ “power” law [27]. In effect, if *F*(*t*) is a subadditive discount function and *G*(*t*) is its corresponding new discount function by using the deformation *D*(*t*) = *t*^*k*^, where *k* > 0, one has *G*(*t*) = *F*(*t*^*k*^). If *k* > 1, a necessary and sufficient condition for *G*(*t*) being an inverse S-curve discount function is that the equation $\frac{k-1}{kt^{k}}=\delta_{V}\left(t^{k}\right)$ has a finite number of solutions, where *δ*_*V*_ is the instantaneous discount rate of the discount function *V*(*t*) ≔ *δ*(*t*)/*δ*(0) (take into account that *δ* is decreasing). Following this methodology, in this manuscript, we have introduced a discounting model where all types of inconsistency are present according to the solutions of the former equation. This particular case is based on the CRDI (constant relative decreasing impatience) discount function proposed by [28, 29]. This class of discount functions generalizes the family of functions introduced by [30] and [31], because they can have every degree of inconsistency.

**Fig 2:**
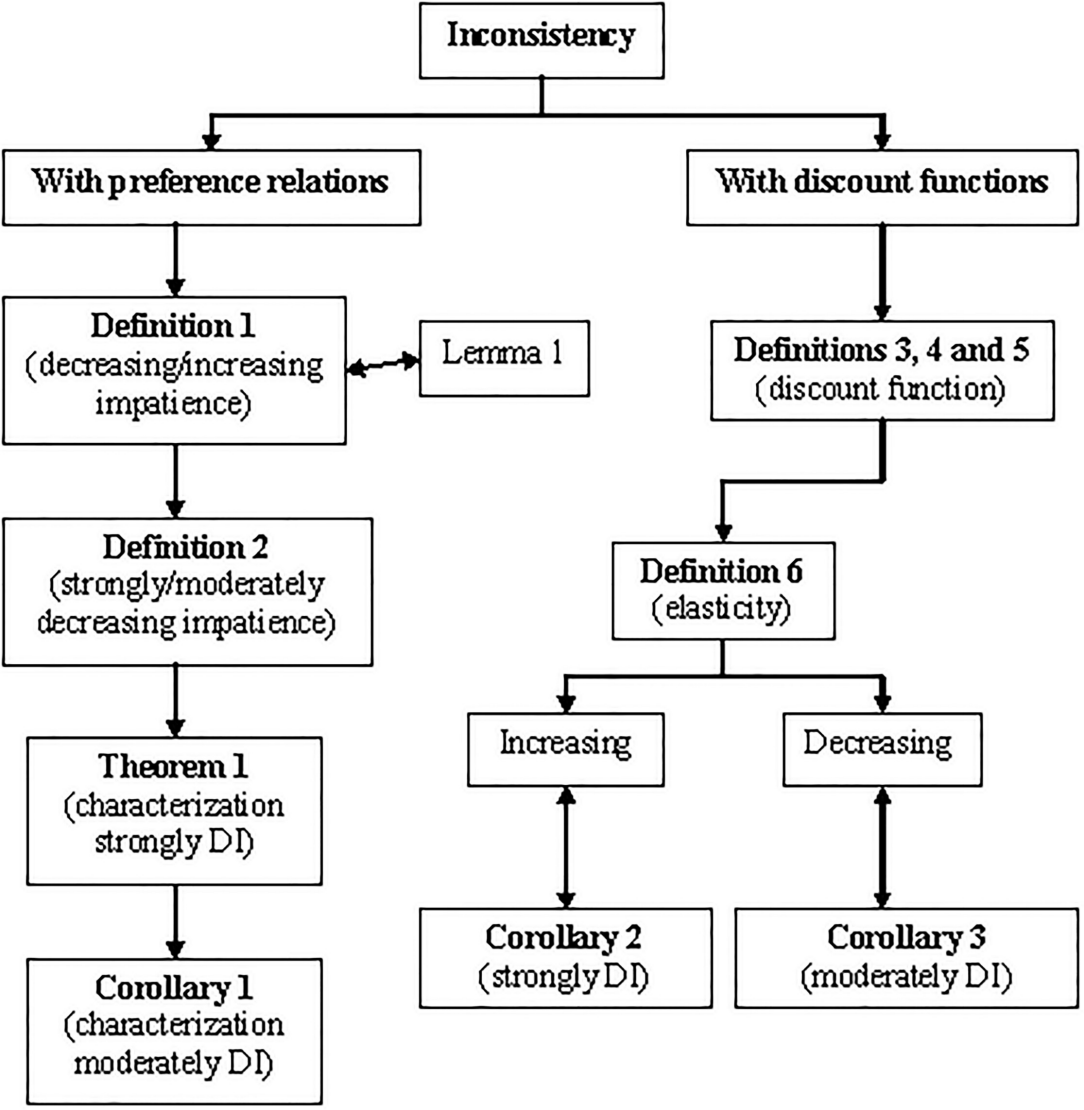
Structure of sections 2 and 4. Source: Own elaboration.

Fig 2 schematizes the contents of this section in order to help readers to follow the development of ideas.

### An approach with preference relations

Let us start with some definitions which will be necessary for the development of this section [32]. Consider the set M=X×T, where *X* = [0, +∞) and *T* = [0, +∞). Let ⪰ be a *preference relation* defined on a subset D⊆M, satisfying:

1. Ordering and continuity, i.e., ⪰ is a continuous, weak (reflexive, transitive and complete) order on D.
2. Monotonicity:
  - For every *s* ∈ *T* and *t* ∈ *T*, then (0, *s*) ∼ (0, *t*).
  - For every *x* ∈ *X*, *y* ∈ *X* and *t* ∈ *T*, then (*x*, *t*) ≻ (*y*, *t*), whenever *x* > *y*.
  - For every *x* ∈ *X*, *s* ∈ *T* and *t* ∈ *T*, then (*x*, *s*) ≻ (*x*, *t*), whenever *s* < *t*.

**Definition 1**. *A decision-making based on preferences* ⪰ *exhibits decreasing (resp. increasing) impatience if, for every* (x,s)∈D *and* (y,t)∈D *such that* (*x*, *s*) ∼ (*y*, *t*) *and* *k* > 0 *such that* (y,t+k)∈D, *one has* (x,s+k)∈D *and* (*x*, *s* + *k*) ≺ (*y*, *t* + *k*) *(resp. (x*, *s* + *k*) ≻ (*y*, *t* + *k*)*)* [11].

Under the conditions of Definition 1, if (*x*, *s* + *k*) ∼ (*y*, *t* + *k*), we will say that the impatience is *constant*. From now on, we will focus on decreasing impatience and assume that $D=X\times [0,t_{0})$ (*t*_0_ can be +∞). Thus, the condition (y,t+k)∈D necessarily implies (x,s+k)∈D. Analogously, in the rest of this section, the requirement (y,t+τ)∈D will imply (x,s+σ)∈D. As a result, the concepts of strongly decreasing impatience and moderately decreasing impatience have been slightly changed with respect to Rohde’s (2015) paper in order to reach more accurate definitions involving the domain of the preference relation and the discount function. With this hypothesis, we can enunciate the following lemma.

**Lemma 1**. *A decision-making based on preferences* ⪰ *exhibits decreasing impatience if, and only if, for every* (x,s)∈D *and* (y,t)∈D *such that* (*x*, *s*) ∼ (*y*, *t*) *and* *τ* > 0 *such that* (y,t+τ)∈D, *there exists* *σ* = *σ*(*x*, *y*, *s*, *t*, *τ*) *(*0 < *σ* < *τ) such that* (x,s+σ)∈D *and* (*x*, *s* + *σ*) ∼ (*y*, *t* + *τ*).

*Proof*. **Necessity**. Let (x,s)∈D and (y,t)∈D be two outcomes such that (*x*, *s*) ∼ (*y*, *t*) and *τ* > 0 such that (y,t+τ)∈D. By hypothesis, one has:

$$\begin{array}{c} (x,s+\tau )≺(y,t+\tau ). \end{array}$$

Moreover, by monotonicity,

$$\begin{array}{c} (y,t+\tau )≺(y,t)∼(x,s). \end{array}$$

Therefore, by transitivity,

$$\begin{array}{c} (x,s+\tau )≺(y,t+\tau )≺(x,s). \end{array}$$

By the continuity of ⪰, there exists *σ* = *σ*(*x*, *y*, *s*, *t*, *τ*) < *τ* such that

$$\begin{array}{c} (x,s+\sigma )∼(y,t+\tau ). \end{array}$$

Finally, as *s* < *t* and *σ* < *τ*, then *s* + *σ* ∈ *T* and so (x,s+σ)∈D.

**Sufficiency**. Reciprocally, let (x,s)∈D and (y,t)∈D be two dated rewards such that (*x*, *s*) ∼ (*y*, *t*) and *k* > 0 such that (y,t+k)∈D. By hypothesis, there exists *h* = *h*(*x*, *y*, *s*, *t*, *k*) (0 < *h* < *k*) such that

$$\begin{array}{c} (x,s+h)∼(y,t+k). \end{array}$$

As (*x*, *s* + *k*) ≺ (*x*, *s* + *h*), by transitivity,

$$\begin{array}{c} (x,s+k)≺(y,t+k), \end{array}$$

as required. Finally, *s* + *k* ∈ *T* and so (x,s+k)∈D.

The following definition was provided by [12, 13, 33].

**Definition 2**. *A decision-making based on preferences* ⪰ *and exhibiting decreasing impatience (σ* < *τ* *in Definition 1) has moderately (resp. strongly) decreasing impatience if* *sτ* < *tσ* *(resp*. *sτ* ≥ *tσ)*.

The following theorem provides a nice characterization of strongly decreasing impatience.

**Theorem 1**. *A decision-making based on preferences* ⪰ *exhibits strongly decreasing impatience if, and only if, for every* (x,s)∈D *and* (y,t)∈D *such that* (*x*, *s*) ∼ (*y*, *t*) and λ > 1 *such that* (y,λt)∈D, *one has* (x,λs)∈D *and* (*x*, λ*s*) ⪯ (*y*, λ*t*).

*Proof*. **Necessity**. Assume first that ⪰ exhibits strongly decreasing impatience. Let (x,s)∈D and (y,t)∈D be two outcomes such that (*x*, *s*) ∼ (*y*, *t*) and λ > 1 such that (y,λt)∈D. We can write (*y*, λ*t*) = (*y*, *t* + (λ − 1)*t*). By Lemma 1, there exists *σ* = *σ*(*x*, *y*, *s*, *t*, λ) (0 < *σ* < (λ − 1)*t*) such that:

$$\begin{array}{c} (x,s+\sigma )∼(y,t+(\lambda -1)t). \end{array}$$

By hypothesis, one has *s*(λ − 1)*t* ≥ *tσ* and, consequently, *s*(λ − 1) ≥ *σ*. Therefore, by monotonicity,

$$\begin{array}{c} \left(x,\lambda s)=(x,s+(\lambda -1)s)⪯(x,\sigma )∼(y,\lambda t\right) \end{array}$$

and, by transitivity, (*x*, λ*s*) ⪯ (*y*, λ*t*). Finally, as *s* < *t*, then λ*s* ∈ *T* and so (x,λs)∈D.

**Sufficiency**. Let (x,s)∈D and (y,t)∈D be two outcomes such that (*x*, *s*) ∼ (*y*, *t*). For every *τ* > 0 such that (y,t+τ)∈D, we can write *t*+*k* ≔ λ*t*, where $\lambda =\frac{t+k}{t}>1$. By hypothesis, (*x*, λ*s*) ⪯ (*y*, λ*t*). Observe that $\lambda s=(1+\frac{k}{t})s<s+k$, from where, by monotonicity,

$$\begin{array}{c} (x,s+k)≺(x,\lambda s)⪯(y,\lambda t). \end{array}$$

Therefore, by transitivity, (*x*, *s* + *k*) ≺ (*y*, *t* + *k*) and so ⪰ exhibits decreasing impatience.

On the other hand, for every *τ* > 0 such that (y,t+τ)∈D, by Lemma 1, there exists *σ* = *σ*(*x*, *y*, *s*, *t*, *τ*) (0 < *σ* < *τ*) such that (x,s+σ)∈D and (*x*, *s* + *σ*) ∼ (*y*, *t* + *τ*). Obviously, there exists λ > 1 such that λ*t* ≔ *t* + *τ*. In effect, it suffices to take $\lambda =1+\frac{\tau }{t}$. By hypothesis,

$$\begin{array}{c} (x,\lambda s)⪯(y,\lambda t)=(y,t+\tau )∼(x,s+\sigma ), \end{array}$$

from where, by monotonicity, λ*s* ≥ *s* + *σ*. Therefore,

$$\begin{array}{c} (1+\frac{\tau }{t})s\geq s+\sigma \end{array}$$

and so *sτ* ≥ *tσ* which means strongly decreasing impatience.

**Fig 3:**
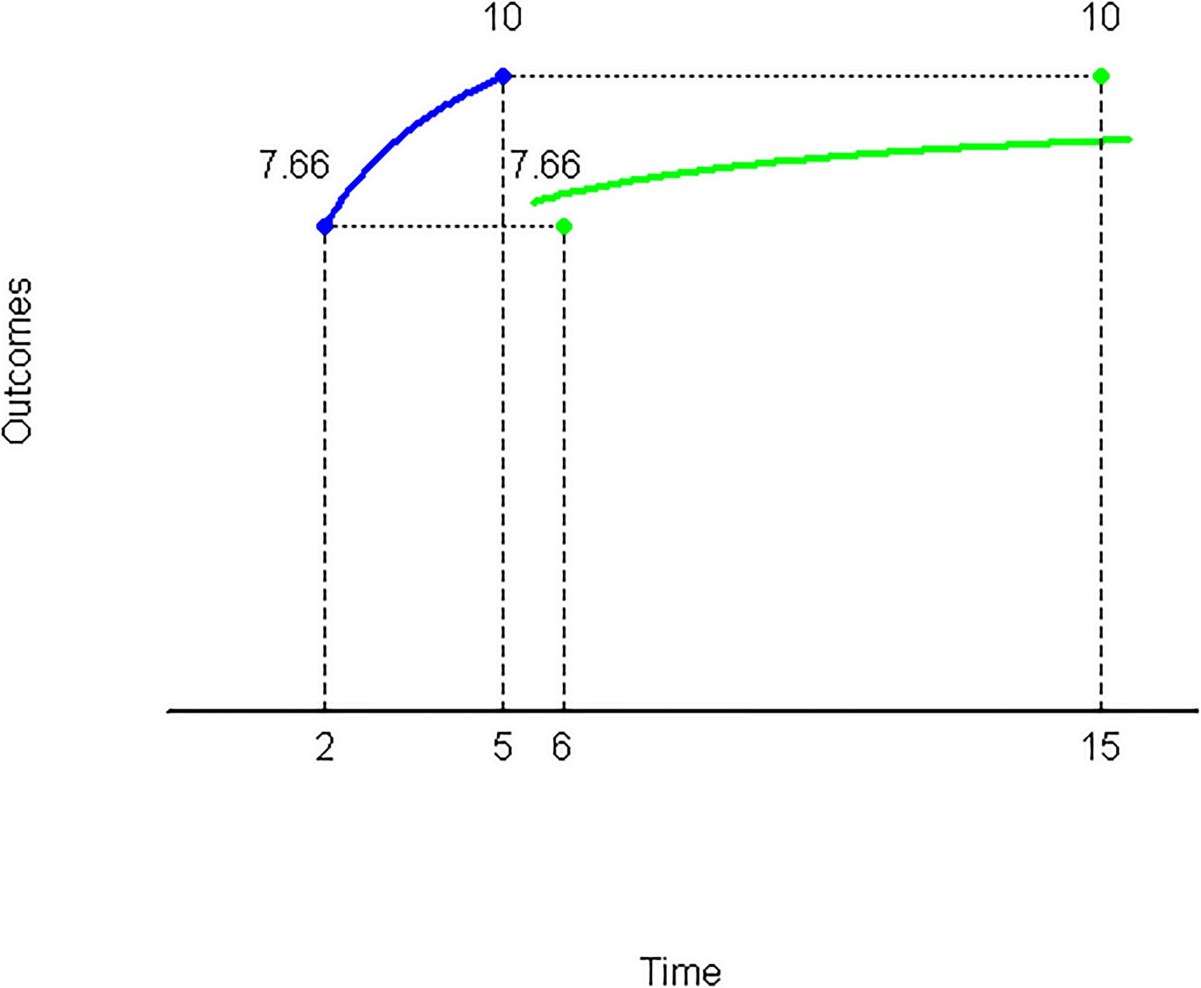
Strongly decreasing impatience.

A graphic representation of Theorem 1 can be observed in Fig 3. In effect, starting from the indifference pair (7.66, 2) ∼ (10, 5) (line in blue), taking the factor λ = 3, one has:

$$\begin{array}{c} \left(7.66,6)⪯(10,15\right) \end{array}$$

(see line in green). Take into account that the rewards above an indifference line are preferred to those below such line.

**Corollary 1**. *A decision-maker exhibiting preferences* ⪰ *has moderately decreasing impatience if, and only if, for every* (x,s)∈D *and* (y,t)∈D *such that* (*x*, *s*) ∼ (*y*, *t*), *k* > 0 *and* λ > 1 *such that* (y,t+k)∈D *and* (y,λt)∈D, *one has* (x,s+k)∈D, (x,λs)∈D *and* (*x*, *s* + *k*) ≺ (*y*, *t* + *k*) *but* (*x*, λ*s*) ≻ (*y*, λ*t*).

**Fig 4:**
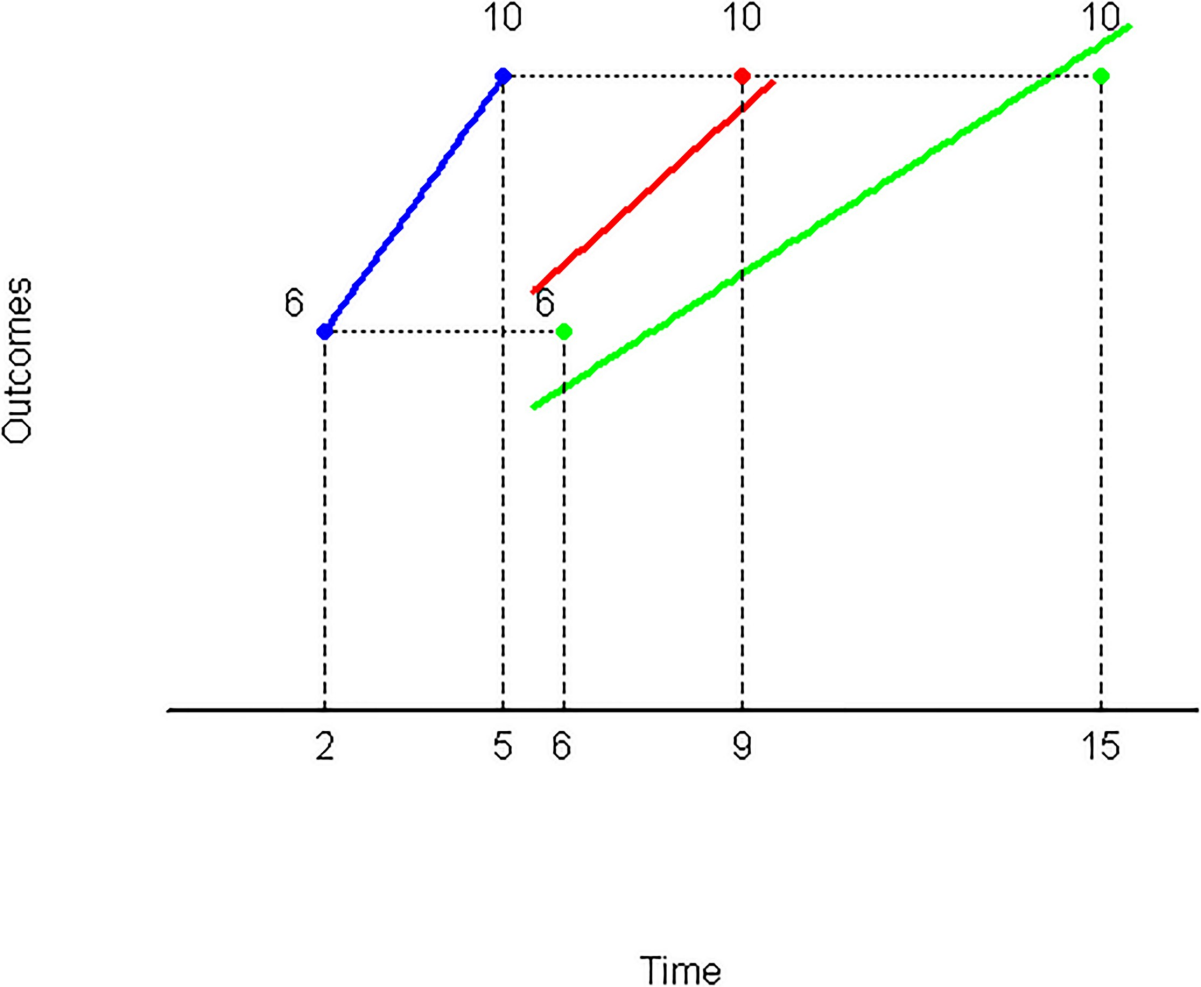
Moderately decreasing impatience.

A graphic representation of Corollary 1 can be observed in Fig 4. In effect, starting from the indifference pair (6, 2) ∼ (10, 5) (line in blue), taking the factor λ = 3, one has:

$$\begin{array}{c} \left(6,6)⪰(10,15\right) \end{array}$$

(see line in green) but, taking the summand *k* = 4, one has:

$$\begin{array}{c} \left(6,6)⪯(10,9\right) \end{array}$$

(see line in red). That is to say, multiplying both dates by λ = 3, the first reward moves above the indifference line, whilst summing up *k* = 4, this reward is now below such line.

### An approach with discount functions

Before starting this section, we need some definitions.

**Definition 3**. *A positive real-valued function*

$$\begin{array}{c} F:D\to R \end{array}$$

*such that*

$$\begin{array}{c} \left(x,t)\mapsto F(x,t\right) \end{array}$$

*is said to be a discount function if F is strictly increasing with respect to x*, *strictly decreasing with respect to t*, *and satisfies*

1. *F*(0, *t*) = 0, *for every* *t* ∈ [0, *t*_0_).
2. *F*(*x*, 0) = *x*, *for every* *x* ∈ *X*.

**Definition 4**. *A discount function F*(*x*, *t*) *is said to be regular if* D=M *and*

$$\begin{array}{c} \lim_{t\to +\infty }F\left(x,t\right)=0. \end{array}$$

*On the other hand*, *F*(*x*, *t*) *is said to be singular if* D=M *and*

$$\begin{array}{c} \lim_{t\to +\infty }F\left(x,t\right)≔L\left(x\right)>0. \end{array}$$

**Example 1**. *The discount function* $F\left(x,t\right)=\frac{x}{1+ixt}$, *i* > 0, *is regular, whilst* $F\left(x,t\right)=x\frac{1+it}{1+jt}$, 0 < *i* < *j*, *is singular*:

$$\begin{array}{c} \lim_{t\to +\infty }F\left(x,t\right)=\frac{i}{j}x. \end{array}$$

There are other discount functions, called “of bounded domain”, such as *F*(*x*, *t*) = *x*(1 − *it*), *i* > 0, whose domain is:

$$\begin{array}{c} D=X\times [0,\frac{1}{i}). \end{array}$$

**Definition 5**. *A discount function* *F*(*x*, *t*) *is said to be separable if* *F*(*x*, *t*) = *u*(*x*)*F*(*t*), *where u is strictly increasing*, *F* *is strictly decreasing*, *u*(0) = 0 *and* *F*(0) = 1.

From now on, we will consider only separable discount functions. In this case, we will be refer to *F*(*t*) as the *unitary discount function* (observe that, in this case, *u*(*x*) = 1). If a separable discount function is regular, then

$$\begin{array}{c} \lim_{t\to +\infty }F\left(t\right)=0. \end{array}$$

On the other hand, if *F*(*t*) is singular, then

$$\begin{array}{c} \lim_{t\to +\infty }F\left(x,t\right)≔L>0. \end{array}$$

The proof of Lemma 1, Theorem 1 and Corollary 1 are also possible by using the separable discount function derived from the preference relation (see Introduction). In effect, the indifference pair (*x*, *s*) ∼ (*y*, *t*) gives rise to the following equality:

$$\begin{array}{c} F\left(s\right)=\frac{y}{x}F\left(t\right). \end{array}$$

For every *τ* > 0 such that (y,t+τ)∈D, the equation in *σ*:

$$\begin{array}{c} F\left(s+\sigma \right)=\frac{y}{x}F\left(t+\tau \right) \end{array}$$

has a solution. In effect, let *L* ≔ lim_*t*→+∞_ *F*(*t*). As $\frac{y}{x}>1$ and *F*(*t* + *τ*) > *L*, then

$$\begin{array}{c} L<\frac{y}{x}F\left(t+\tau \right)<\frac{y}{x}F\left(t\right)=F\left(s\right). \end{array}$$

Therefore, by continuity, there exists *σ* > 0 such that

$$\begin{array}{c} F\left(s+\sigma \right)=\frac{y}{x}F\left(t+\tau \right). \end{array}$$

In order to present the following two results which characterize both strongly and moderately decreasing impatience, we need the following definition.

**Definition 6**. *Let* *F*(*t*) *be a unitary discount function, differentiable in its domain*. *The elasticity of* *F*(*t*) *is defined by*:

$$\begin{array}{c} \epsilon \left(t\right)≔t\frac{F^{\prime }\left(t\right)}{F(t)}=t{\left(\ln F\right)}^{\prime }\left(t\right)=-t\delta \left(t\right). \end{array}$$ (7)

**Corollary 2**. *A decision-maker exhibiting preferences governed by a separable discount function* *F*(*x*, *t*) = *u*(*x*)*F*(*t*) *has strongly decreasing impatience if, and only if* *ϵ*(*t*) *is increasing*.

*Proof*. **Necessity**. Let *r* ∈ *T* and *s* ∈ *T* be two instants such that *r* < *s*. As *X* = [0, , +∞[, we can find two outcomes *v* and *w* such that

$$\begin{array}{c} u(v)F(r)=u(w)F(s). \end{array}$$ (8)

By Theorem 1, for every λ > 1 such that (w,λs)∈D, one has (v,λr)∈D and (*v*, λ*r*) ⪯ (*w*, λ*s*), from where

$$\begin{array}{c} u(v)F(\lambda r)\leq u(w)F(\lambda s). \end{array}$$ (9)

By dividing the two left-hand sides and the two right-hand sides of Eq (8) and inequality (9), we obtain:

$$\begin{array}{c} \frac{F(\lambda r)}{F(r)}\leq \frac{F(\lambda s)}{F(s)} \end{array}$$

and so, taking Napierian logarithms,

$$\begin{array}{c} \ln F(\lambda r)-\ln F(r)\leq \ln F(\lambda s)-\ln F(s). \end{array}$$

Observe that the former inequality can be written as follows:

$$\begin{array}{c} \frac{r}{s}\frac{\ln F(r+(\lambda -1)r)-\ln F(r)}{(\lambda -1)r}\leq \frac{\ln F(s+(\lambda -1)s)-\ln F(s)}{(\lambda -1)s}. \end{array}$$

Letting λ → 1, one has:

$$\begin{array}{c} \frac{r}{s}{\left(\ln F\right)}^{\prime }\left(r\right)\leq {\left(\ln F\right)}^{\prime }\left(s\right), \end{array}$$

from where

$$-r{\left(\text{ln}\;F\right)}^{\prime }\left(r\right)\;\geq \;-\;s{\left(\text{ln}\;F\right)}^{\prime }\left(s\right),$$

which means that *ϵ*(*t*) is decreasing.

**Sufficiency**. It is obvious.

**Corollary 3**. *A decision-maker exhibiting preferences governed by a separable discount function* *F*(*x*, *t*) = *u*(*x*)*F*(*t*) *has moderately decreasing impatience if*, *and only if*, *ϵ*(*t*) *is increasing*.

*Proof*. In effect, if *ϵ* is increasing, then

$$\begin{array}{c} \epsilon^{\prime }\left(t\right)=-\delta \left(t\right)-t\delta^{\prime }\left(t\right)>0, \end{array}$$

which necessarily requires that *δ*′(*t*) < 0. Therefore, the decision-maker exhibits decreasing impatience. The rest of the proof is analogous to that Corollary 2.

## Searching a new measure of inconsistency

The index of convexity introduced by Prelec [11]:

$$\begin{array}{c} P\left(t\right)≔-\frac{{\left(\ln F\right)}^{\prime \prime }\left(t\right)}{{\left(\ln F\right)}^{\prime }\left(t\right)}=-\frac{\delta^{\prime }\left(t\right)}{\delta (t)}=-{\left(\ln\delta \right)}^{\prime }\left(t\right) \end{array}$$ (10)

is not able to distinguish if the discount function *F*(*t*) exhibits moderately or strongly decreasing impatience (see Table 1). Our aim is to derive a new index able to detect these changes of decreasing impatience, apart from increasing impatience.

**Definition 7**. *For every indifference* (*x*, *s*) ∼ (*y*, *t*), *the hyperbolic factor is defined by*:

$$\begin{array}{c} H\left(s,t,\sigma ,\tau \right)≔\frac{\tau -\sigma }{t\sigma -s\tau }, \end{array}$$ (11)

*provided that*

$$\begin{array}{c} (x,s+\sigma )∼(y,t+\tau ). \end{array}$$

If the decision-maker exhibits increasing impatience, then *τ* < *σ* and so *sτ* < *tσ*. Consequently, *H*(*s*, *t*, *σ*, *τ*) < 0. In case of decreasing impatience, the *τ* > *σ* and so *H*(*s*, *t*, *σ*, *τ*) > 0 is the decision-maker exhibits moderately decreasing impatience, and *H*(*s*, *t*, *σ*, *τ*) < 0, otherwise.

By dividing both the numerator and the denominator of *H*(*s*, *t*, *σ*, *τ*) (Eq (11)) by *τ*, one has:

$$\begin{array}{c} H\left(s,t,\sigma ,\tau \right)≔\frac{1-\frac{\sigma }{\tau }}{t\frac{\sigma }{\tau }-s}. \end{array}$$

Taking the limit of *H*(*s*, *t*, *σ*, *τ*) when *τ* converges to zero (which implies *σ* → 0), as [10]

$$\begin{array}{c} \lim_{\tau \to 0}\frac{\sigma }{\tau }=\frac{\delta (t)}{\delta (s)}, \end{array}$$

one has:

$$\begin{array}{c} \lim_{\tau \to 0}H\left(s,t,\sigma ,\tau \right)≔H\left(s,t\right)=\frac{1-\frac{\delta (t)}{\delta (s)}}{t\frac{\delta (t)}{\delta (s)}-s}=\frac{\frac{\delta (s)-\delta (t)}{\delta (s)}}{\frac{t\delta (t)-s\delta (s)}{\delta (s)}}=\frac{\frac{\delta (s)-\delta (t)}{t-s}}{\frac{t\delta (t)-s\delta (s)}{t-s}}. \end{array}$$

Taking now the limit of *H*(*s*, *t*) when *s* converges to *t*,

$$\begin{array}{c} \lim_{s\to t}H\left(s,t\right)≔H\left(t\right)=\frac{-\delta^{\prime }\left(t\right)}{{\left[t\delta \left(t\right)\right]}^{\prime }}=\frac{\delta^{\prime }\left(t\right)}{\epsilon^{\prime }\left(t\right)}. \end{array}$$ (12)

**Example 2**. *Analyze the impatience exhibited by the discount function*

$$\begin{array}{c} F(t)=\exp\{\exp\{-kt\}-1\},\;\;\;k>0. \end{array}$$

*In this case*,

- *δ*(*t*) = *k* exp{−*kt*}, *and*
- *ϵ*(*t*) = −*kt* exp{−*kt*}.

*By differentiating both functions*,

- *δ*′(*t*) = −*k*^2^ exp{−*kt*} *(so* *F*(*t*) *exhibits decreasing impatience), and*
- *ϵ*′(*t*) = −*k* exp{−*kt*}+*k*^2^*t* exp{−*kt*}.

*Therefore, by simplifying both the numerator and the denominator of the expression of* *H*(*t*), *one has*:

$$\begin{array}{c} H\left(t\right)=\frac{k}{1-kt}. \end{array}$$

*Thus, for example, if* *k* = 0.3, *H*(*t*) *is positive for* *t* < 3.33, *and negative, for* *t* > 3.33. *In other words*, *F*(*t*) *exhibits moderately decreasing impatience for* *t* < 3.33 *and shows strongly decreasing impatience for* *t* > 3.33. *Observe that this result coincides with the conclusion of Example 2 in* [14].

**Example 3**. *Analyze the impatience exhibited by the discount function*

$$\begin{array}{c} F(t)=\exp\{-\arctan(t)\}. \end{array}$$

*In this case*,

- $\delta \left(t\right)=\frac{1}{1+t^{2}}$, *and*
- $\epsilon \left(t\right)=-\frac{t}{1+t^{2}}$.

*By differentiating both functions*,

- $\delta^{\prime }\left(t\right)=-\frac{2t}{{\left(1+t^{2}\right)}^{2}}$ *(so* *F*(*t*) *exhibits decreasing impatience), and*
- $\epsilon^{\prime }\left(t\right)=\frac{-1+t^{2}}{{\left(1+t^{2}\right)}^{2}}$.

*Therefore, by simplifying both the numerator and the denominator of the expression of* *H*(*t*), *one has*:

$$\begin{array}{c} H\left(t\right)=\frac{-2t}{-1+t^{2}}. \end{array}$$

*Thus*, *F*(*t*) *exhibits strongly decreasing impatience for long term periods* *(t* > 1 *)* *(see* Fig 3). *Observe that this result coincides with the conclusion of Example 3 in* [14].

**Proposition 1**. *H*(*t*) *is constant if, and only if*, *F*(*t*) *is the* *q-exponential discount function*.

*Proof*. In effect, let *F*(*t*) be the *q*-exponential discount function [34]:

$$\begin{array}{c} F\left(t\right)=\frac{1}{{\left[1+\left(1-q\right)kt\right]}^{1/(1-q)}},\;\;\;k>0,\;\;\;q\in R\backslash \left\{1\right\}. \end{array}$$ (13)

In this case,

- $\delta \left(t\right)=\frac{k}{1+(1-q)kt}$, and
- $\epsilon \left(t\right)=-\frac{kt}{1+(1-q)kt}$.

By differentiating both functions,

- $\delta^{\prime }\left(t\right)=-\frac{\left(1-q\right)k^{2}}{{\left[1+\left(1-q\right)kt\right]}^{2}}$, and
- $\epsilon^{\prime }\left(t\right)=-\frac{k}{{\left[1+\left(1-q\right)kt\right]}^{2}}$.

Therefore, by simplifying both the numerator and the denominator of the expression of *H*(*t*), one has:

H(t)=(1-q)k

and so *H*(*t*) is constant.

Reciprocally, if *H*(*t*) is constant:

$$\begin{array}{c} H(t)=h \end{array}$$

or, equivalently,

$$\begin{array}{c} \delta^{\prime }\left(t\right)=h\epsilon^{\prime }\left(t\right). \end{array}$$

By integrating both hand sides of the former equality, one has the following chain of equalities:

$$\begin{array}{c} \delta (t)=h\epsilon (t)+k, \end{array}$$

$$\begin{array}{c} \delta (t)=-ht\delta (t)+k, \end{array}$$

$$\begin{array}{c} \delta (t)(1+ht)=k, \end{array}$$

from where:

$$\begin{array}{c} \delta \left(t\right)=\frac{k}{1+ht}. \end{array}$$

Therefore,

$$\begin{array}{c} F\left(t\right)=\frac{1}{{\left(1+ht\right)}^{k/h}}. \end{array}$$

Making *k*/*h* equal to 1/(1 − *q*), we obtain *h* = *k*(1 − *q*) and so

$$\begin{array}{c} F\left(t\right)=\frac{1}{{\left[1+\left(1-q\right)kt\right]}^{1/(1-q)}}, \end{array}$$

which is the *q*-exponential discount function. Finally, observe that *k* = *δ*(0) > 0.

In what follows, we are going to analyze the sign and the possible values of *H*(*t*). To do this, we can distinguish three cases:

1. *δ*′(*t*) > −*ϵ*′(*t*). In this case, we can consider the following three possibilities:
  - If *F*(*t*) exhibits increasing impatience, then *δ*′(*t*) > 0 and *H*(*t*) < 0, whereby *ϵ*′(*t*) < 0 and so:
    $$\begin{array}{c} \delta^{\prime }\left(t\right)>-\epsilon^{\prime }\left(t\right)>0. \end{array}$$
    Therefore,
    $$\begin{array}{c} H(t)<-1. \end{array}$$
  - If *F*(*t*) exhibits strongly decreasing impatience, then *δ*′(*t*) < 0 and *H*(*t*) < 0, whereby *ϵ*′(*t*) > 0 and so:
    $$\begin{array}{c} -\epsilon^{\prime }\left(t\right)<\delta^{\prime }\left(t\right)<0. \end{array}$$
    Therefore,
    $$\begin{array}{c} -1<H(t)<0. \end{array}$$
  - Finally, if *F*(*t*) exhibits moderately decreasing impatience, then
    $$\begin{array}{c} H(t)>0. \end{array}$$
2. *δ*′(*t*) < −*ϵ*′(*t*). In this case, we can consider the following three possibilities:
  - If *F*(*t*) exhibits increasing impatience, then *δ*′(*t*) > 0 and *H*(*t*) < 0, whereby *ϵ*′(*t*)<0 and so:
    $$\begin{array}{c} 0<\delta^{\prime }\left(t\right)<-\epsilon^{\prime }\left(t\right). \end{array}$$
    Therefore,
    $$\begin{array}{c} -1<H(t)<0. \end{array}$$
  - If *F*(*t*) exhibits strongly decreasing impatience, then *δ*′(*t*) < 0 and *H*(*t*) < 0, whereby *ϵ*′(*t*) > 0 and so:
    $$\begin{array}{c} \delta^{\prime }\left(t\right)<-\epsilon^{\prime }\left(t\right)<0. \end{array}$$
    Therefore,
    $$\begin{array}{c} H(t)<-1. \end{array}$$
  - Finally, if *F*(*t*) exhibits moderately decreasing impatience, then
    $$\begin{array}{c} H(t)>0. \end{array}$$
3. *δ*′(*t*) = −*ϵ*′(*t*). In this case, *h* = 1 in Proposition 1 and, consequently, *F*(*t*) = (1 − *t*)^*k*^, *k* > 0 which, obviously, exhibits increasing impatience.

By putting cases 1 and 3 together, Tables 2 and 3 summarize the obtained results.

**Table 2:** Types of impatience according to the values of *H*(*t*) (*δ*′(*t*)≥ −*ϵ*′(*t*)). Source: Own elaboration.

Increasing Impatience	Strongly DI	Constant Impatience	Moderately DI
*H*(*t*) ≤ −1	−1 < *H*(*t*) < 0	*H*(*t*) = 0	*H*(*t*) > 0

**Table 3:** Types of impatience according to the values of *H*(*t*) (*δ*′(*t*) < −*ϵ*′(*t*)). Source: Own elaboration.

Strongly DI	Increasing Impatience	Constant Impatience	Moderately DI
*H*(*t*) < −1	−1 < *H*(*t*) < 0	*H*(*t*) = 0	*H*(*t*) > 0

**Corollary 4**. *The q-exponential discount function can not exhibit strongly decreasing impatience*.

*Proof*. In effect, taking into account Proposition 1, *ϵ*′(*t*) < 0, from which:

- If *q* < 1, then *δ*′(*t*) < 0 and *H*(*t*) > 0. Therefore, *F*(*t*) exhibits moderately decreasing impatience.
- If *q* → 1, *F*(*t*) tends to the exponential discounting which shows constant impatience.
- If *q* > 1, then *δ*′(*t*) > 0 and *H*(*t*) < 0. Therefore, *F*(*t*) exhibits increasing impatience.

The former result can be confirmed by Fig 4 in which the indifference lines have been derived from the hyperbolic discounting (specifically, $F\left(t\right)=\frac{1}{1+0.4t}$). Definitively, in order to unify the former two cases in only one and that the new index has the same sign as Prelec’s measure, we re going to introduce the following definition:

**Definition 8**. *Let* *F*(*t*) *be a discount function*. *The instantaneous impatience index of* *F*(*t*) *at time* *t*, *denoted by* *I*^3^(*t*), *is given by*:

$$\begin{array}{c} I^{3}\left(t\right)=1+{\left[\frac{\delta^{\prime }\left(t\right)}{\epsilon^{\prime }\left(t\right)}\right]}^{\text{sign}\left[\delta^{\prime }\left(t\right)+\epsilon^{\prime }\left(t\right)\right]}. \end{array}$$ (14)

Table 4 exhibits the range of values of *I*^3^(*t*). Observe that *I*^3^(*t*) has the same sign as *P*(*t*), depending on whether *F*(*t*) exhibits increasing or decreasing impatience.

**Table 4:** Types of impatience according to the values of *I*^3^(*t*) and *P*(*t*). Source: Own elaboration.

Increasing Impatience	Strongly DI	Constant impatience	Moderately DI
*I*^3^(*t*) ≤ 0	0 < *I*^3^(*t*) < 1	*I*^3^(*t*) = 1	*I*^3^(*t*) > 1
*P*(*t*) ≤ 0	*P*(*t*) > 0		

As indicated, the instantaneous impatience index generalizes Prelec’s index. In effect, assume that sign[*δ*′(*t*) + *ϵ*′(*t*)] = +1. In this case,

$$\begin{array}{c} I^{3}\left(t\right)=1+\frac{\delta^{\prime }\left(t\right)}{\epsilon^{\prime }\left(t\right)}=\frac{\delta^{\prime }\left(t\right)+\epsilon^{\prime }\left(t\right)}{\epsilon^{\prime }\left(t\right)}. \end{array}$$

Obviously, the numerator is positive. In case of decreasing impatience, then *δ*′(*t*) < 0. On the other hand, if the numerator has to be positive, necessarily *ϵ*′(*t*) > 0 holds. Therefore, *I*^3^(*t*) > 0. In case of increasing impatience, then *δ*′(*t*) > 0. On the other hand, *ϵ*′(*t*) = −*δ*(*t*) − *tδ*′(*t*) < 0 holds. Therefore, *I*^3^(*t*) < 0.

Analogously, assume that sign[*δ*′(*t*) + *ϵ*′(*t*)] = −1. In this case,

$$\begin{array}{c} I^{3}\left(t\right)=1+\frac{\epsilon^{\prime }\left(t\right)}{\delta^{\prime }\left(t\right)}=\frac{\delta^{\prime }\left(t\right)+\epsilon^{\prime }\left(t\right)}{\delta^{\prime }\left(t\right)}. \end{array}$$

Obviously, the numerator is negative. In case of decreasing impatience, then *δ*′(*t*) < 0 and so *I*^3^(*t*) > 0. On the other hand, in case of increasing impatience, then *δ*′(*t*) > 0 and so *I*^3^(*t*) < 0.

In consequence, *I*^3^(*t*) has the same sign as *P*(*t*) which means an improvement when measuring inconsistency.

## Analysis of a special case: The S-inverse discount functions

Let *F*(*t*) be a subadditive discount function which exhibits decreasing impatience and *k* a real number greater than 1. Consider the new discount function defined by:

$$\begin{array}{c} G\left(t\right)≔F\left(t^{k}\right). \end{array}$$ (15)

It can be shown that:

- *δ*_*G*_(*t*) = *kt*^*k*−1^ *δ*(*t*^*k*^).
- *ϵ*_*G*_(*t*) = −*kt*^*k*^ *δ*(*t*^*k*^).

On the other hand,

- $\delta_{G}^{\prime }\left(t\right)=k\left(k-1\right)t^{k-2}\delta \left(t^{k}\right)+k^{2}t^{2(k-1)}\delta^{\prime }\left(t^{k}\right)$. Observe that $\delta_{G}^{\prime }\left(t\right)$ is not necessarily positive because *δ*′(*t*^*k*^) is negative.
- $\epsilon_{G}^{\prime }\left(t\right)=-k^{2}t^{k-1}\delta \left(t^{k}\right)-k^{2}t^{2k-1}\delta^{\prime }\left(t^{k}\right)$.

Therefore,

$$\begin{array}{c} H_{G}\left(t\right)=\frac{\left(k-1\right)\delta \left(t^{k}\right)+kt^{k}\delta^{\prime }\left(t^{k}\right)}{-kt\delta \left(t^{k}\right)-kt^{k+1}\delta^{\prime }\left(t^{k}\right)}. \end{array}$$ (16)

Observe that, if *δ*′(0) < +∞, *H*_*G*_(*t*) can be written as:

$$\begin{array}{c} H_{G}\left(t\right)=\frac{\left(k-1\right)-kt^{k}\delta_{V}\left(t^{k}\right)}{-kt+kt^{k+1}\delta_{V}\left(t^{k}\right)}, \end{array}$$ (17)

where *V*(*t*) is the discount function defined as:

$$\begin{array}{c} V\left(t\right)≔\frac{\delta (t)}{\delta (0)}, \end{array}$$ (18)

which is well defined because *δ*(0) > 0.

Now, we are going to consider the zeros and poles of *H*(*t*). A possible solution of equation $\delta_{G}^{\prime }\left(t\right)=0$, denoted by *t*_0_, must satisfy:

$$\begin{array}{c} \delta_{V}\left(t_{0}^{k}\right)=\frac{k-1}{kt_{0}^{k}}, \end{array}$$ (19)

whilst a possible solution of equation $\epsilon_{G}^{\prime }=0$, denoted by *t*_1_, must satisfy:

$$\begin{array}{c} \delta_{V}\left(t_{1}^{k}\right)=\frac{1}{t_{1}^{k}}. \end{array}$$ (20)

Observe that the existence of *t*_1_ implies the existence of *t*_0_. In effect, the functions

$$\begin{array}{c} m\left(t\right)≔\frac{k-1}{kt^{k}}=\frac{1-\frac{1}{k}}{t^{k}} \end{array}$$

and

$$\begin{array}{c} n\left(t\right)≔\frac{1}{t^{k}} \end{array}$$

are decreasing and *m*(*t*) < *n*(*t*) which necessarily implies 0 < *t*_0_ < *t*_1_. In this context, we are assuming that both intersections giving rise to *t*_0_ and *t*_1_ are not secant. So we can distinguish the following three cases:

1. 0 < *t* < *t*_0_. In this case, the preference exhibits increasing impatience.
2. *t*_0_ < *t* < *t*_1_. In this case, the preference exhibits moderately decreasing impatience.
3. *t*_1_ ≤ *t*. In this case, the preference exhibits strongly decreasing impatience.

Finally, observe that more situations are possible, depending on the existence of more solutions of the former two equations (which depends on the shape of *δ*_*V*_(*t*^*k*^)).

**Example 4.** *Consider the CRDI (constant relative decreasing impatience) discount function* [28]:

$$\begin{array}{c} F(t)=\exp\{\exp\{-ct\}-1\},\;\;\;c>0. \end{array}$$

*This function exhibits decreasing impatience and so we can consider the deformation of time by means of its k-th power, giving rise to the following new discount function*:

$$\begin{array}{c} G\left(t\right)=\exp\{\exp\left\{-ct^{k}\right\}-1\},\;\;\;c>0,\;\;\;k>1. \end{array}$$

*In this case*,

$$\begin{array}{c} H_{G}\left(t\right)=\frac{\left(k-1\right)-ckt^{k}}{-kt+ckt^{k+1}}. \end{array}$$

*Therefore*,

$$\begin{array}{c} t_{0}={\left(\frac{k-1}{ck}\right)}^{1/k} \end{array}$$

*and*

$$\begin{array}{c} t_{1}={\left(\frac{1}{c}\right)}^{1/k}, \end{array}$$

*and so we can distinguish the following three cases*:

1. 0 < *t* < *t*_0_. *In this case*, *the preference exhibits increasing impatience*.
2. *t*_0_ < *t* < *t*_1_. *In this case*, *the preference exhibits moderately decreasing impatience*.
3. *t*_1_ ≤ *t*. *In this case*, *the preference exhibits strongly decreasing impatience*.

**Fig 5:**
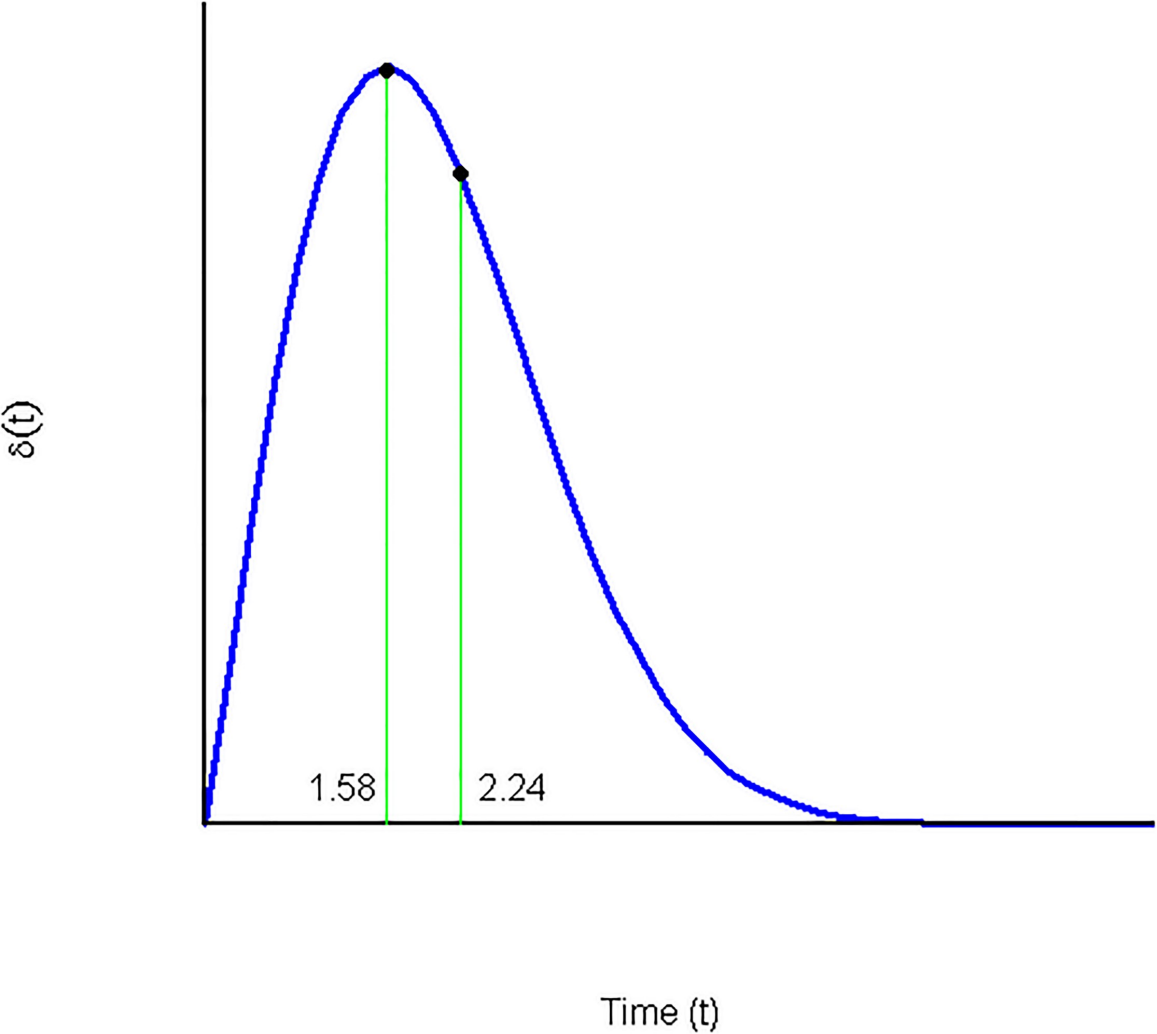
Discount rate of a deformation of the CRDI discount function.

*More specifically*, *for the concrete values* *c* = 0.2 *and* *k* = 2, *one has (see* Fig 5*)*:

1. *If* 0 < *t* < 1.58, *the preference exhibits increasing impatience*.
2. *If* 1.58 < *t* < 2.24, *the preference exhibits moderately decreasing impatience*.
3. *If* 2.24 ≤ *t*, *the preference exhibits strongly decreasing impatience*.

**Fig 6:**
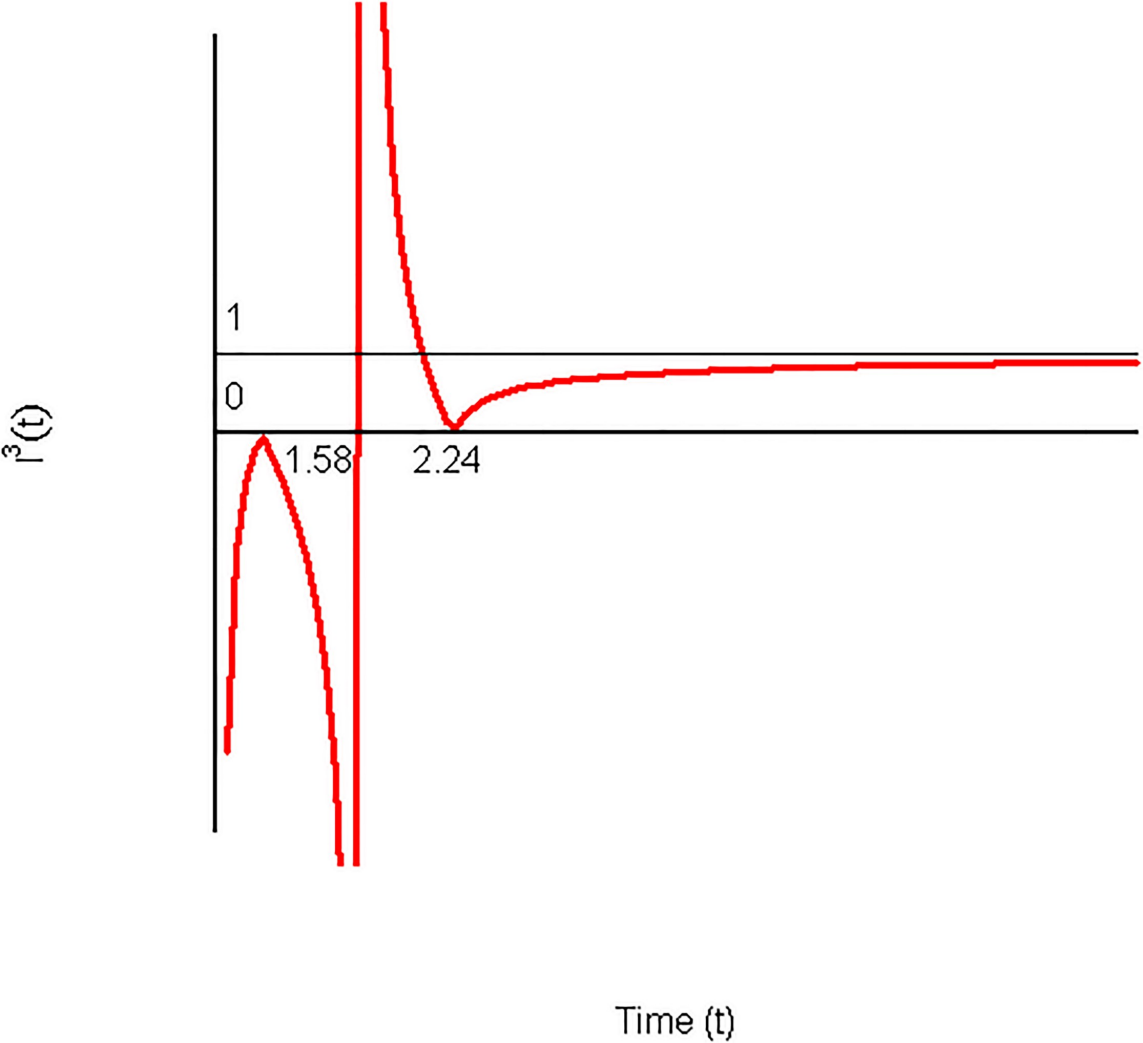
Instantaneous impatience index corresponding to Fig 5.

*Observe that the instantaneous discount rate of* *G*(*t*) *increases from 0 to 1.58, later there exists a moderate decrease until 2.24 and, finally, the rest of the discount rate decrease is strong*. *These results can be confirmed by the graphic representation of* *I*^3^(*t*) *in* Fig 6 *which corroborates the results obtained in* Fig 5 *in the way that, from 0 to 1.58*, *I*^3^(*t*) < 0, *between 1.58 and 2.24*, 0 < *I*^3^(*t*) < 1, *and finally after 2.28*, *I*^3^(*t*) > 1 *(see* Table 4*)*.

## Conclusions

This paper dealt with the topic of inconsistency in intertemporal choice by distinguishing between increasing and decreasing impatience. Within this last group, we will follow the classification provided by [35] who discriminates between moderately and strongly decreasing impatience. According to the treatment of inconsistency with preferences, we have obtained nice characterizations of increasing impatience, moderately decreasing impatience and strongly decreasing impatience by using constant and proportional increase of time, and the tools of differential calculus. Finally, the main contribution of this manuscript is the introduction of a novel index (denoted by *I*^3^(*t*)) which can distinguish the intervals in which a discount function exhibits either increasing impatience or moderately or strongly decreasing impatience. Indeed, this index improves the DI-index (and then Prelec’s measure of inconsistency) which only discriminates between increasing and decreasing impatience, and also improves the hyperbolic factor which exhibits the same sign for increasing and strongly decreasing impatience.

As a further research, we would like to design a suitable survey in order to analyze the degree of inconsistency *I*^3^(*t*) exhibited by several groups of subjects [36, 37] of different age [38, 39], sex, marital status, purchasing power, country, etc.

## References

[1] Cruz Rambaud S, Muñoz Torrecillas MJ. Measuring impatience in intertemporal choice. PLoS ONE 2016 2;11(2):e0149256 10.1371/journal.pone.0149256 26890895 PMC4758727

[2] Fishburn PC, Rubinstein A. Time preference. International Economic Review 1982 10;23(3):677–694. 10.2307/2526382

[3] Samuelson PA. A note on measurement of utility. The Review of Economic Studies 1937 2;4(2):155–161. 10.2307/2967612

[4] Takahashi T, Han R, Nakamura F. Time discounting: Psychophysics of intertemporal and probabilistic choices. Journal of Behavioral Economics and Finance 2012 6;5:10–14.

[5] Kirby KN, Herrnstein R. Preference reversals due to myopic discounting of delayed reward. Psychological Science 1995 3;6(2):83–90. 10.1111/j.1467-9280.1995.tb00311.x

[6] Kirby KN. Bidding on the future: Evidence against normative discounting of delayed rewards. Journal of Experimental Psychology: General 1997 3;126(1):54–70. 10.1037/0096-3445.126.1.54

[7] Strotz R. Myopia and inconsistency in dynamic utility maximization. Review of Economic Studies 1955 12;23(3):165–180. 10.2307/2295722

[8] Cruz Rambaud S, González Fernández I, Ventre V. Modeling the inconsistency in intertemporal choice: The generalized Weibull discount function andits extension. Annals of Finance 2018 8;14(3):415–426. 10.1007/s10436-018-0318-3

[9] Cruz Rambaud S, Ventre A.G.S. Intertemporal choice and nonadditive capitalization functions. International Journal of Intelligent Systems 2011 1;26(1):63–72. 10.1002/int.20453

[10] Cruz Rambaud S, Muñoz Torrecillas MJ. Capitalization speed of a financial law. Proceedings of the Fourth Italian-Spanish Conference on FinancialMathematics. Alghero (Italy) from June 28 to July 1, 2001.

[11] Prelec D. Decreasing impatience: A criterion for non-stationary timepreference and “hyperbolic” discounting. Scandinavian Journal of Economics 2004 9;106(3):511–532. 10.1111/j.0347-0520.2004.00375.x

[12] Rohde KIM. The hyperbolic factor: A measure of time inconsistency. Journal of Risk and Uncertainty 2010 10;41(2):125–140. 10.1007/s11166-010-9100-2

[13] Rohde KIM. An index to measure decreasing impatience. Working paper of the Tinbergen Institute and Erasmus Research Instituteof Management 2015.

[14] González Fernández I, Cruz Rambaud S. Inconsistency in intertemporal choice: A behavioral approach. European Journal of Management and Business Economics 2018 10;27(3):231–248. 10.1108/EJMBE-01-2018-0012

[15] Haushofer J, Cornelisse S, Seinstra M, Fehr E, Joëls M, Kalenscher T. No effects of psychosocial stress on intertemporal choice. PLoS One 2013 11;8(11):e78597 10.1371/journal.pone.0078597 24250800 PMC3826744

[16] Bickel WK, Jarmolowicz DP, Mueller ET, Koffarnus MN, Gatchalian KM. Excessive discounting of delayed reinforcers as a transdisease process contributing to addiction and otherdisease-related vulnerabilities: Emerging evidence. Pharmacology and Therapeutics 2012 6;134(3):287–297. 10.1016/j.pharmthera.2012.02.004 22387232 PMC3329584

[17] MacKillop J, Amlung MT, Few LR, Ray LA, Sweet LH, Munafò MR. Delayed reward discounting and addictive behavior: A meta-analysis. Psychopharmacology 2011 8;216(3):305–321. 10.1007/s00213-011-2229-0 21373791 PMC3201846

[19] Snider SE, DeHart WB, Epstein LH, Bickel WK. Does delay discounting predict maladaptive health and financial behaviors in smokers?. Health Psychology 2019 1;38(1):21–28. 10.1037/hea0000695 30474996 PMC6601630

[18] Bickel WK, Odum AL, Madden GJ. Impulsivity and cigarette smoking: Delay discounting in current, never, and ex-smokers. Psychopharmacology 1999 10;146(4):447–454. 10.1007/pl00005490 10550495

[20] Madden GJ, Petry NM, Badger GJ, Bickel WK. Impulsive and self-control choices in opioid-dependent patients and non-drug-usingcontrol participants: Drug and monetary rewards. Experimental and Clinical Psychopharmacology 1997 8;5(3):256–262. 10.1037/1064-1297.5.3.256 9260073

[21] Coffey SF, Gudleski GD, Saladin ME, Brady KT. Impulsivity and rapid discounting of delayed hypothetical rewards in cocaine-dependent individuals. Experimental and Clinical Psychopharmacology 2003 2;11(1):18–25. 10.1037/1064-1297.11.1.18 12622340

[22] Petry NM. Discounting of delayed rewards in substance abusers: Relationship to antisocial personality disorder. Psychopharmacology 2002 8;162(4):425–432. 10.1007/s00213-002-1115-1 12172697

[23] Cruz Rambaud S, Muñoz Torrecillas MJ, Takahashi T. Observed and normative discount functions in addiction and other diseases. Frontiers in Pharmacology 2017 6;8(416):1–10. 28706486 10.3389/fphar.2017.00416 PMC5489713

[24] Sayman S, Öncüler A. An investigation of time inconsistency. Management Science 2009 3;55(3):470–482. 10.1287/mnsc.1080.0942

[25] Takeuchi K. Non-parametric test of time consistency: Present bias and future bias. Games and Economic Behavior 2011 3;71:456–478. 10.1016/j.geb.2010.05.005

[26] Dos Santos LS, Martinez AS. Inconsistency and subjective time dilation perception in intertemporal decision making. Frontiers in Applied Mathematics and Statistics 2018 11;4:54 10.3389/fams.2018.00054

[27] Cruz Rambaud S, Ventre V. Deforming time in a nonadditive discount function. International Journal of Intelligent Systems 2016 9;32(5):467–480. 10.1002/int.21842

[28] Bleichrodt H, Rohde KIM, Wakker PP. Non-hyperbolic time inconsistency. Games and Economic Behavior 2009 5;6:27–38. 10.1016/j.geb.2008.05.007

[29] Bleichrodt H, Gao Y, Rohde KIM. A measurement of decreasing impatience for health and money. Journal of Risk and Uncertainty 2016 9;52(3):213–231. 10.1007/s11166-016-9240-0

[30] Prelec D. Decreasing impatience: Definition and consequences. Harvard Business School Working Paper no. 90-015 1989.

[31] Ebert JEJ, Prelec D. The fragility of time: Time-insensitivity and valuation of the near and far future. Management Science 2007 7;53(9):1423–1438. 10.1287/mnsc.1060.0671

[32] Baucells M, Heukamp FH. Probability and time trade-off. Management Science 2012 4;58(4):831–842. 10.1287/mnsc.1110.1450

[33] Rohde KIM. Decreasing relative impatience. Journal of Economic Psychology 2009 12;30(6):831–839. 10.1016/j.joep.2009.08.008

[34] Cruz Rambaud S, Muñoz Torrecillas MJ. A generalization of the *q*-exponential discounting function. Physica A: Statistical Mechanics and its Applications 2013 7;392(14):3045–3050. 10.1016/j.physa.2013.03.009

[35] Rohde KIM. Measuring decreasing and increasing impatience. Management Science 2019 4;65(4):1455–1947. 10.1287/mnsc.2017.3015

[36] Guo Q-W, Chen S, Schonfeld P, Li Z. How time-inconsistent preferences affect investment timing for rail transit. Transportation Research Part B 2018 10;118:172–192. 10.1016/j.trb.2018.10.009

[37] Millner A, Heal G. Time consistency and time invariance in collective intertemporal choice. Journal of Economic Theory 2018 3;176:158–169. 10.1016/j.jet.2018.03.002

[38] Lemoine D. Age-induced acceleration of time: Implications for intertemporal choice. Journal of Economic Behavior and Organization 2018 7;153:143–152. 10.1016/j.jebo.2018.07.002

[39] Löckenhoff CE, Samanez-Larkin GR. Age differences in intertemporal choice:The role of task type, outcome characteristics, and covariates. Journals of Gerontology. Series B: Psychological Sciences & Social Sciences 2019 8;gbz097. 10.1093/geronb/gbz097 PMC6909431 31410482

